# Digital screening of children with ASD: diagnostic accuracy of emotion recognition and visual preference tasks

**DOI:** 10.1186/s12888-025-07725-z

**Published:** 2025-12-25

**Authors:** Larissa Pliska, Isabel Neitzel, Michael Buschermöhle, Olga Kunina-Habenicht, Ute Ritterfeld

**Affiliations:** 1 TU Dortmund University, Emil-Figge-Straße 50, 44227 Dortmund, Germany; 2 KIZMO GmbH, Steinweg 13-17, 26122 Oldenburg, Germany

**Keywords:** Autism spectrum disorder, Emotion recognition, Visual preference, Digital screening, Diagnostic accuracy, Sensitivity, Specificity

## Abstract

**Background:**

In Germany, there is a need to improve care for suspected autism spectrum disorder (ASD) cases, as the time between parents’ initial suspicion and an official clinical diagnosis can reach three years. New technologies for digital screening promise relief in addressing children’s difficulties in recognizing emotions and social attention. This study investigated the diagnostic validity of tablet-based screening to differentiate between children with and without ASD via emotion recognition and visual preference tasks involving prior calibration.

**Method:**

This study involved 24 boys with ASD and a matched control group of 24 typically developing (TD) boys aged 6–11 years. Mixed logistic models were applied for the emotion recognition task, while mixed linear models were used for the visual preference task, along with decision trees for both tasks.

**Results:**

The results indicate significant group differences in recognizing the emotion “fear” and naming an example for “sadness”. The emotion recognition of fear and sadness was relevant to the decision tree to differentiate between the groups, with an accuracy of 81.25%, a sensitivity of 91.67%, and a specificity of 70.83%. For the visual preference task, no significant group differences were found between groups; however, significant differences emerged between social and non-social image stimuli. Gaze fixation and gaze changes in video stimuli were relevant to the decision tree to differentiate between the groups. The accuracy was 81.25%, with a sensitivity of 70.83% and specificity of 91.67%.

**Conclusion:**

Overall, this study suggests that automated digital screening might provide support and relief to families and clinicians, as it can distinguish between children with and without ASD using a combination of selected emotion recognition and visual preference tasks.

**Clinical trial number:**

Not applicable.

**Supplementary Information:**

The online version contains supplementary material available at 10.1186/s12888-025-07725-z.

## Introduction

Autism spectrum disorder (ASD) is a neurodevelopmental disorder [1] with a worldwide prevalence of approximately 1 in 100 children [2]. More recent data from the Autism and Developmental Disabilities Monitoring Network shows that, in 2022, around 1 in 31 eight-year-olds had ASD [3]. Although the clinical presentation of ASD is heterogeneous and symptoms vary from person to person and are multifaceted [4], the main symptoms of ASD are long-term deficits in social interaction and communication as well as restricted interests and repetitive behaviors [1]. The impairment of social interaction includes non-verbal communication behaviors, including the ability to read and signal emotions [5]. Thus, people with ASD may have difficulties recognizing faces and emotions [6]. For example, a meta-analysis by Uljarevic and Hamilton [7] reported difficulties with emotion recognition in individuals with ASD, with a mean effect size of 0.80 (0.41 when corrected for publication bias). Another symptom is the so-called abnormal social attention of children with ASD [8, 9], which can be used to understand deficits in social interaction [9]. Abnormal social attention refers to the visual preference for non-social stimuli over social stimuli [9]. For example, a meta-analysis by Chita-Tegmark [10] showed that individuals with ASD spend less time attending to social stimuli than typically developing (TD) individuals do, with a mean effect size of 0.55.

Existing diagnostic tools have focused on deviated patterns of social communication and interaction to diagnose ASD [11]. However, the process of diagnosing ASD is characterized by delays and long waiting times. It can take up to three years from the onset of symptoms and the parents’ initial concerns to obtain a diagnosis [12]. In Germany, for example, parents typically report concerns about their child’s behavior around the age of two years, yet the average age of the first diagnosis is 6;6 years, when most children are already attending school [13]. This significant delay can be explained by two challenges. First, the rapidly increasing demand for early detection and diagnosis may contribute to delayed ASD diagnoses, as this time-consuming process relies on a limited number of well-trained practitioners [14]. In addition, the increasing prevalence of ASD [14] has put extra demand on clinicians, with this increase being likely a result of greater public awareness and progress in case detection through significant improvements in early identification. Although the final diagnosis using gold standard measures cannot be replaced by an automated diagnostic tool, an early screening of the likelihood of ASD in a child could reduce the workload of clinicians by decreasing the number of children on the waiting list who are no longer suspected of having ASD after screening. Second, the symptoms of ASD are highly heterogeneous, which complicates the diagnostic process [15]. Such delays have detrimental effects on individuals and families seeking proper diagnoses [12]. Not only do they create uncertainty for the entire family [16], they also delay the initiation of family support and child therapy [17]. Given that an early start to intervention has been shown to lead to better developmental outcomes, this delay may result in additional adverse effects [16, 18]. This clearly demonstrates the need for improved forms of care for suspected cases of ASD – not only, but especially in Germany (see [13]).

Digital technologies have the potential to support the screening and diagnostic process in ASD [19, 20], making clinical decisions more objective and reducing necessary resources [21]. Digital screening can be an effective method for detecting symptoms of ASD; it can be conducted by non-specialists and may take place in the child’s natural environment [19, 22]. Digital technologies have already been used in a number of studies to identify people at risk of ASD effectively (overview in [22]). One example is the early autism screening tool (EAST), an interactive tablet-based app that assesses preschool children in Pakistan on how they relate to people, imitation, motor skills, visual, and intellectual responses through play-based activities in home, school, or clinical settings [23]. Another example is ASDetect, a free mobile application based on the Social Attention and Communication Surveillance (SACS) tool, which assesses behavioral markers of ASD from 12 to 24 months of age [24]. In addition, a web-based online screening tool called the Web Italian Network for Autism Spectrum Disorder (WIN4ASD), an innovative web application for pediatricians, was developed in Italy [25]. Perochon et al. [26] reported on a digital autism screening application (app) that displays stimuli that elicit behavioral signs of ASD, quantified via computer vision and machine learning. Another current example is the German IDEAS project^1^. IDEAS stands for ‘Identification of Autism Spectrum Disorder using speech and facial expression recognition’. This project involves developing a tablet-based digital screening tool (app). Since the gold standard instrument for diagnosing ASD (Autism Diagnostic Observation Schedule, Second Edition; ADOS-2; [27]) uses a combination of tasks due to the heterogeneity of ASD symptoms, this should also be taken into account in digital screening. Therefore, exploratory analyses are necessary to examine individual tasks that may discriminate between children with ASD and TD in combination. In this context, our study aims to investigate whether prototype tasks on emotion recognition and visual preference for this tablet-based screening can effectively discriminate between children with and without ASD. It is therefore necessary to check the diagnostic accuracy and combination of these two tasks.

### Emotion recognition

Emotional impairment, including deficits in emotion recognition, has been proposed as a diagnostic criterion for ASD [28]. In general, emotion recognition refers to the attribution of emotional states by analyzing non-verbal cues, such as facial expressions [29]. Emotion recognition between the ages of 6 and 11 years is a predictor of well-being and social relationships [30]. However, the literature shows contradictory effects of emotion recognition in people with ASD [7].

For example, a study by Castelli [31] showed that children with ASD were just as proficient as TD children at recognizing all six basic emotions (fear, happiness, anger, sadness, disgust, surprise; [32]) from facial expressions. However, it should be noted that, at the beginning, emotion recognition was practiced and describing the emotion was also considered correct (for example, for the emotion sadness, it was also considered correct if the child said ‘crying’), which is why no difference was found. In an untimed condition, in which stimuli were presented until the participant responded, Nagy et al. [33] also showed that children with and without ASD were equally accurate in identifying emotions. Only in the timed condition, when stimuli were shown for a maximum of 1,200 ms, were the children with ASD less accurate at identifying anger and surprise [33]. In the study by Tanaka et al. [34], the children were shown images expressing an emotion with different emotion labels until one label was selected. This may explain why their study found no differences in basic emotion recognition between children with and without ASD, except for anger [34]. In addition, the results of a meta-analysis by Icht et al. [35] also suggest that people with ASD do not differ from TD individuals in the recognition of simple prosodic emotions such as sadness and happiness.

In contrast, other studies have shown that people with ASD display poorer emotion recognition than TD individuals do [36] and that emotion recognition is poorer in individuals with ASD than in individuals in other clinical groups [37]. Meta-analyses by Uljarevic and Hamilton [7], by Lozier et al. [6] and by Yeung [37] analyzed emotion recognition in individuals with ASD and reported a significant impairment in the recognition of basic emotions in individuals with ASD. However, recognition of happiness was only slightly impaired in individuals with ASD, and fear was only slightly less frequently recognized correctly than happiness [7]. In a study by Evers et al. [38], in addition to some emotion-specific impairments in children with ASD aged 6–14 years, a generally poorer performance in emotion recognition compared with TD children was described. However, the participants showed a preference for the selection of certain emotion labels. Fear was selected more often by children with ASD than TD children. This could also explain the better performance of children with ASD in identifying fear. As a result of this preference, the data were corrected for response bias. Afterwards, the results no longer show any emotion-specific impairment but still show an overall lower emotion recognition performance in individuals with ASD [38]. Compared with the findings of Evers et al. [38], a synthesis of data from 72 papers by Leung et al. [39] demonstrated that the accuracy of emotion recognition is generally impaired in people with ASD but also that people with ASD display longer response times for a subset of emotions (anger, fear, sadness vs. performance across all six basic emotions). Findings by Griffiths et al. [40] provide further evidence of an impairment in the recognition of basic emotions in children and adolescents with ASD. For all basic emotions, the difference in emotion recognition is significant. One reason for these differences could be the sex ratio between the groups (ASD group predominantly boys, control group balanced sex ratio; [40]), as females have been shown to perform better on emotion recognition tasks [41]. However, the accuracy of recognizing the emotion “fear” was low in both groups, which can be attributed to an overall poorer recognition of this emotion in both groups [40]. This contradicts the reported preference for fear reported by Evers et al. [38].

Song and Hakoda [42] also addressed the recognition thresholds at which children with and without ASD correctly identified emotional expressions that changed from a neutral expression to the corresponding basic emotion. Overall, they reported that the different emotions had different recognition thresholds in both groups. However, compared with TD children, children with ASD generally require a significantly greater intensity of stimuli to correctly identify anger, disgust, and fear. These results suggest a selective impairment rather than a general impairment in the recognition of basic emotions [42].

There is no difference in emotion recognition between older children with ASD and adults with ASD [43]. In addition, between the ages of 8 and 12 years, children with ASD appear to catch up with TD children at this age, so that by the age of 12 years, children with and without ASD no longer differ in the recognition of basic emotions, and this remains comparable to adolescence. Findings by Rump et al. [43] show, on the one hand, that children with ASD between the ages of 5 and 7 years recognized some emotions less accurately than TD children did and, on the other hand, that children with ASD recognized emotions differently in general. TD children were significantly better at recognizing fear and anger according to the study results. However, there was no significant difference in the recognition of happiness and sadness. Overall, happiness was the easiest emotion for both groups to recognize, whereas children with ASD had significantly more difficulties recognizing anger and fear [43].

However, the ability to recognize one’s own emotions (emotional self-awareness) may be essential for recognizing the emotions of others (cf [44]). In addition to deficits in emotion recognition, people with ASD are described as having difficulties with emotional self-awareness, i.e., recognizing and understanding their own feelings [45]. In a meta-analysis by Huggins et al. [45], 32 out of 41 studies reported significantly lower emotional self-awareness in people with ASD. Meta-analyses of self-report measures have shown that people with ASD have significantly impaired overall emotional self-awareness (*d* = 1.16). However, concerning age groups, differences in emotional self-awareness appear only in adolescents (*d* = 0.63; 12 years and younger: *d* = 0.03) and increase with age (*d* = 1.16 to *d* = 1.58; 45).

In summary, research shows contradictory results as to whether children with ASD perform poorer at recognizing basic emotions than children without ASD [7, 31, 36, 39, 40]. Given the inconsistent results regarding emotion recognition, it is important to examine whether this task can distinguish between children with and without ASD in a digital setting. It would also be interesting to establish whether only certain emotions can be used for this purpose. There is evidence that differences in emotion recognition depend on the emotion [38, 43], and it has been described that children with ASD have problems with emotional self-awareness [45]. An emotion recognition task involving the description of a situation in which an emotion is experienced may thus be a more decisive task for screening to differentiate ASD from TD individuals. Therefore, we investigate the emotion recognition task in an appropriate digital task setting that includes two parts. The first part of this study addresses the following research question:

1. Can an emotion recognition task (comprising emotion recognition and situation description) be used to distinguish between children with and without ASD?

### Visual preference

One of the most reliable measures for discriminating between the ASD and TD groups was a reduced preference for social stimuli [22]. Such a task can be implemented via readily available technology (e.g., tablets) in conjunction with objective performance measurements, making it usable by non-specialists [22]. To measure children’s attentional preferences for social versus non-social stimuli, a promising screening approach is to use eye-tracking technology [46]. Eye-tracking technologies can be considered complementary to traditional screening measures [19]. A meta-analysis by Chita-Tegmark [10] examined social attention in studies using eye-tracking to compare people with and without ASD and evaluated factors that might influence effect sizes. The results show that, with a mean effect size of 0.55, people with ASD spend significantly less time paying attention to social stimuli than TD people do. Social attention^2^ is most affected in people with ASD when stimuli have high social content (more than one person; [10]).

In a study by Wang et al. [47], human eyes and high-/low-autism-interest objects were presented simultaneously to children. Participants with and without ASD showed reduced social preference when an image of human eyes was paired with objects attributed to high ASD interest (cars; [47]). However, it would be oversimplifying to assume that all individuals with ASD should have a special interest in a particular object. Therefore, these designs are debatable and can be seen as limitations of these studies. Nevertheless, findings by Ambarchi et al. [8] suggest atypical attentional patterns in social and non-social domains in ASD, with ASD interests playing a reduced role. Ambarchi et al. [8] reported that children with ASD are significantly slower in prioritizing faces than TD children are. Similarly, a study by Goold et al. [48] revealed that adults with ASD-associated traits show a faster saccadic response to shift attention away from a face and to recognize the non-social target than adults with low levels of ASD-associated traits do. In both studies [8, 48], faces displaying different emotional expressions were used instead of neutral faces. However, Chawarska et al. [49] demonstrated that six-month-old infants who were later diagnosed with ASD in childhood paid significantly less attention to a social scene and spent significantly less time looking at the actress in general and at her neutral face in particular while watching the scene, compared to control groups.

Crawford et al. [50] investigated whether attentional allocation was influenced by potential threats, i.e., whether stimuli were moving toward or by an observer. The results indicated that adolescents with ASD had a shorter gaze duration for social videos compared to non-social videos only when the stimuli were moving toward them [50]. In addition, an automated eye-tracking task by Strathearn et al. [51] showed that children and adolescents with ASD had a stronger gaze preference for visually organized and structured images than TD controls did. Similarly, a study by Cardon and Azuma [52] noted that the visual attention of children with ASD can be influenced by the type of presentation, as the children showed a visual preference for video presentations over live presentations. However, the result was also evident in the TD group, and it should be noted that both groups consisted of only nine children each.

A study by Król and Król [53] revealed that the sequence of eye movements (saccades) of participants with ASD was significantly greater when they responded to non-social images and significantly lower when they responded to social images than TD individuals did. In addition, the sequence of eye movements in participants with ASD was more similar to that in TD participants when they responded to non-social images and less similar when they responded to social images. The results suggest that individuals with ASD have an attentional bias for non-social stimuli and a weaker preference for social stimuli [53].

In terms of visual preference in children with ASD, Kou et al. [54] addressed three different stimulus paradigms. The first paradigm included dancing people versus dynamic geometric images, which was the most effective scenario. In this paradigm, children with ASD showed a significantly greater preference for dynamic geometric images than for dancing people. A study by Robain et al. [55], with a similar design to the first paradigm, supported the findings. Furthermore, visual preference in 4- to 6-year-old children with ASD appears to be modulated by the type of visual stimulus [56]. Thus, the results of a study by Shi et al. [56] show that, in comparison with TD children, children with ASD pay less attention to stimuli with two or more children playing together than to stimuli with only one child. The second paradigm involves the biological motion of points of light versus non-biological motion of points of light [54]. Similar to the results of Kou et al. [54], Kaliukhovich et al. [57] reported that participants with ASD spent significantly less time looking at presented stimuli than controls did and showed less preference for biological motion. For the third paradigm – a toy alone vs. a child playing with that toy – group differences were also found between the ASD group and the TD group [54].

Chevallier et al. [58] investigated the social attention of children with ASD compared with that of TD children via three different tasks: (1) static visual exploration (images of people vs. images of objects), (2) dynamic visual exploration (videos of people vs. videos of objects), and (3) interactive visual exploration (videos of children playing with objects). Significant differences concerning social attention in the ASD group were exclusively found in the third task (interactive visual exploration). Reduced social attention for children with ASD was evident here [58]. However, a study by Cilia et al. [59] showed that, in addition to dynamic stimuli, static stimuli are also relevant for differentiating between children with ASD and TD children.

Pliska et al. [60] investigated visual preference in boys aged 6–11 years with and without ASD in terms of stimulus presentation (images vs. video) and stimulus complexity (one child playing alone vs. two children playing together). This study revealed no significant differences in visual preference. Neither the format of stimulus presentation nor the different levels of stimulus complexity had any effect on visual preference. However, participants with ASD had already received ASD-specific therapy, which may have had a significant impact on the results [60]. On the other hand, a study by Fischer et al. [61] also found no differences in visual attention between children with ASD and TD children aged 5–12 years. Matyjek et al. [62] conducted a study of visual preference for social and geometric motion in adults with and without ASD. The difference between the adults was in the duration of fixation. Adults without ASD spent more time looking at social stimuli than at geometric movements. In contrast, there were no differences in fixation between social and geometric stimuli in adults with ASD [62]. Overall, the findings of a study by Matyjek et al. [62] suggest that the decisive aspect is not whether children with ASD look at non-social stimuli more than do TD children, which has been the focus of previous studies [9, 54, 63, 64], but whether there is a difference in preference between social and non-social stimuli for TD children and no difference in preference for children with ASD. Overall, it can therefore be assumed that there are differences in visual preferences between people with and without ASD, but that these may vary by age group (infants, children, adults). Therefore, the second part of this study addresses the following research question:

2. Can a visual preference task be used to distinguish between children with and without ASD?

## Method

### Participants

A total sample of 48 children aged 6–11 years was included in the current study. Although the long-term goal of this study is to contribute to the early identification of ASD, the age range of 6–11 years was chosen for an initial exploratory investigation. For this initial investigation, it is important to ensure that the children in the ASD group have a confirmed ASD diagnosis, which on average is achieved at 6;6 years of age in Germany [13]. Data for this study were collected from February 2023 to March 2025. Participants were recruited through various autism therapy centers in the area, open all-day schools, and private contacts. All participating families were informed in writing about the purpose and procedure of the study and they provided written consent. The children were verbally informed again at the testing appointment and gave their consent. In addition, a positive ethics vote for the study was obtained in advance (GEKTUDO_2022_45). This study was not pre-registered. The study design included two groups: (1) the ASD group and (2) the TD group. The two groups were matched for children’s age, sex, and parents’ level of education. The prerequisites for participation in the study were that the children could speak fluently and appropriately for their age (as assessed by their parents in the TD group and ASD therapists in the ASD group), and that they had adequate hearing. Most parents in both groups hold a high school diploma (median score). Given that the ratio of males to females diagnosed with ASD is approximately 3:1 [65] and that the symptoms of females are thought to be qualitatively different from those of boys [66], only boys were included in the study for comparability.

The ASD group consisted of 24 boys who had previously been officially diagnosed with ASD and exhibited no intellectual impairment. Of those boys, 20 boys had already received ASD-specific therapy. In Germany, a diagnosis of ASD is a prerequisite for receiving ASD-specific therapy. ASD is typically diagnosed using a combination of the Autism Diagnostic Observation Schedule and the revised Autism Diagnostic Interview [67] according to the ICD-10 classification system. The children with ASD were, on average, 112.17 months (9;4 years) old (*SD* = 21.65) and had received, on average, 13.67 months (*SD* = 11.67; range = 0–48 months) of therapy. The German version of the Social Communication Questionnaire (FSK; [68]) was used to confirm that the children in the TD group were below the cut-off score for suspected ASD, while those in the ASD group were above it due to the ASD-specific support they had already received. The FSK is a screening instrument that follows the diagnostic guidelines of the ICD-10 [68]. All children in the ASD group had a diagnosis of ASD, although 8 out of 24 children no longer met the screening threshold for a suspected diagnosis of ASD (≥ 16) based on the FSK [68] – completed by parents – at the time of participation in the study. The duration of therapy was negatively correlated with the FSK score (*r* = -.23), but this correlation was not significant (*p* = .323). This result suggests that the longer children receive ASD-specific support, the fewer symptoms of ASD their parents perceive, which is an essential goal of such support or therapy programs. Five children reported attention-deficit (hyperactivity) disorder (AD(H)D) in addition to ASD, and none of them were taking medication during the tasks. AD(H)D is often comorbid with ASD [69]. One child also has impulse control and a reactive attachment disorder. Two other children displayed an impulse control disorder as well as ASD, and one of them had additional verbal developmental dyspraxia. Three children had tic disorders, and one also had anxiety and attachment disorder. Tics were not exhibited during the task. In addition, one child had epilepsy, one had a sleep disorder, and another had a perceptual disorder. Information about additional disorders in addition to ASD is based solely on information provided by the parents. Six children wore glasses.

The TD group consisted of 24 TD boys aged 109.96 months on average (*SD* = 19.78), which was equal to 9 years and 2 months. All children in the TD group scored below the FSK screening threshold for suspected ASD (< 16). The parents of this group reported that two children had ADHD, one had a reading and spelling disorder and one had a tic disorder. Three children wore glasses.

### Measures

This study was part of the IDEAS research project, which aimed to develop an automated digital screening for ASD. The tasks were completed by all the participants in two different conditions: with a human experimenter and with a pre-recorded avatar experimenter (details in [70]). The results of a first analysis by Pliska et al. [71] suggest that children with ASD interact in the same way (significant equivalence) regardless of the experimenter’s digital mediation, and there is no need to distinguish between the two conditions in the analysis. The same is assumed for the control group, although this has not been empirically tested. The tasks were presented to the participating children on a 25-inch screen positioned approximately 60 cm in front of them. The children who needed glasses wore them throughout the entire study.

#### Emotion recognition task

The emotion recognition task used pictures of children with facial expressions from the Child Emotion Facial Expression Set by Negrão et al. [30]. Five pictures of a girl showing the following basic emotions were used: happiness, anger, sadness, disgust, and fear. The test administrators selected this child’s pictures because they judged that the child in the stimulus set most clearly expressed the emotions depicted in these pictures. This selection was made independently of gender.

The emotion of surprise was excluded from the study because previous studies (e.g [72, 73]). have called into question whether surprise is a basic emotion. The participants were always shown one picture on a screen with the question “How does the child feel?”. After the response of the participants to this question, the experimenter named the emotion and asked when the participant had experienced that emotion before, e.g., in the case of happiness: “This is happiness. The child is happy. When are you happy?”. In the following, the participants should describe a situation in which they experienced this emotion.

#### Visual preference task

The visual preference task consisted of different stimulus presentation formats (images vs. videos) and different levels of stimulus complexity (one person vs. two people). The participants saw pairs of social and non-social stimuli side by side on a screen. It was systematically counterbalanced, whether the social stimulus or the non-social stimulus was shown on the left or right side of the screen. The participants saw a total of six pairs of approximately 10s in duration each, with the social image showing one or more people playing and the non-social image showing images of the universe (galaxies and stars). In addition, four pairs of videos of approximately 18s were used, with the social video showing a man or woman making dynamic grimaces and the non-social video showing dynamic geometric shapes. This video material stems from a study by Polzer et al. [74]. During the presentation of the image and video pairs, eye movements (i.e., eye position and gaze direction) were captured and recorded with up to six frames per second via a customized native iOS app (KIZMO Face-Analyzer). The storage rate of six frames per second was selected because it was important to record where participants looked for longer periods of time (several seconds), while short-term saccades were irrelevant. This app was developed by KIZMO GmbH for use on iPads, specifically for this task, and it was developed based on a person wearing glasses. The iPad was placed behind the screen, with the camera to the left of the screen during the task. Prior to the visual preference task, a calibration was performed to relate eye movements to gaze direction in screen coordinates.

### Data analysis

R Studio version 4.4.2 [75] was used for data analysis. The ‘lmerTest’ package [76] was used for random effects models, the ‘emmeans’ package [77] was used for post-hoc tests, and the ‘MuMIn’ package [78] was used for variance components. Plots were generated via the “ggplot2” package [79]. The packages “rpart” [80], “party” [81], “ggparty” [82], and “rpart.plot” [83] were used to calculate accuracy, sensitivity, and specificity and to plot the decision trees. A significance level of *p* < .05 was used in all calculations.

#### Emotion recognition task

Logistic mixed models were used to analyze the emotion task. The dependent variable was once emotion recognition, coded 0 for not recognizing the emotion and 1 for correctly recognizing the emotion, and once experienced emotion example, coded 0 for no specific example and 1 for a specific example. The fixed effects were group, emotion, and the interaction of the two, and the random effects were child ID. Post-hoc tests were performed with Bonferroni correction.

To test whether the length of therapy had an effect on the dependent variable in relation to emotions, models were also created for the ASD group. Decision trees were computed with the dependent variable “group” (ASD and TD) and potential predictors of recognition of happiness, of anger, of sadness, of disgust, and of fear, as well as naming a specific example of happiness, of anger, of sadness, of disgust, and of fear. Therefore, the Gini index, a common measure of inequality [84] and a popular attribute selection measure for decision trees [85], was used.

#### Visual preference task

Based on the previous calibration, the raw data from the AR kit is converted into screen coordinates. These transformed viewpoints are then used during measurement and analysis. The relative percentage of frames recorded in the left and right image/video recordings (social or non-social) was analyzed via MATLAB [86]. On the basis of presentation (social or non-social), the percentage of frames per medium (image or video) was then summed and averaged. In addition, the number of gaze changes between the left and right stimuli was recorded.

Linear mixed models were used to analyze the visual preference task. The dependent variable was gaze fixation duration, and the main effects and interactions were (1) group, presentation, and medium; (2) medium, presentation, and FSK; and (3) group, presentation, and age. The random effects were child ID and stimulus pairs. In another model, the dependent variable was the number of gaze changes, and the presentation was omitted from the fixed effects. Decision trees with the Gini index were computed with the dependent variable “group” (ASD and TD) and potential predictors of gaze fixation of social image, of non-social image, of social video, and of non-social video, as well as gaze change of medium image, and of medium video.

#### Comparison of decision trees

A decision tree for the emotion recognition task and a decision tree for the visual preference task were each cross-tabulated with the predictions. These results were then compared with those of the actual groups.

## Results

### Emotion recognition task

The two groups correctly recognized each emotion with varying frequency, except for happiness (see Supplement Figure S1). The emotion “happiness” was correctly recognized by 75% of the participants in both groups. Fear was correctly recognized by 17% of the TD group and by 65% of the ASD group. The emotion “disgust” was correctly recognized by 38% of the TD group and by 33% of the ASD group. Sadness was correctly recognized by 88% of the TD group and by 57% of the ASD group. The emotion “anger” was correctly recognized by 75% of the TD group and by 57% of the ASD group.

**Fig. 1:**
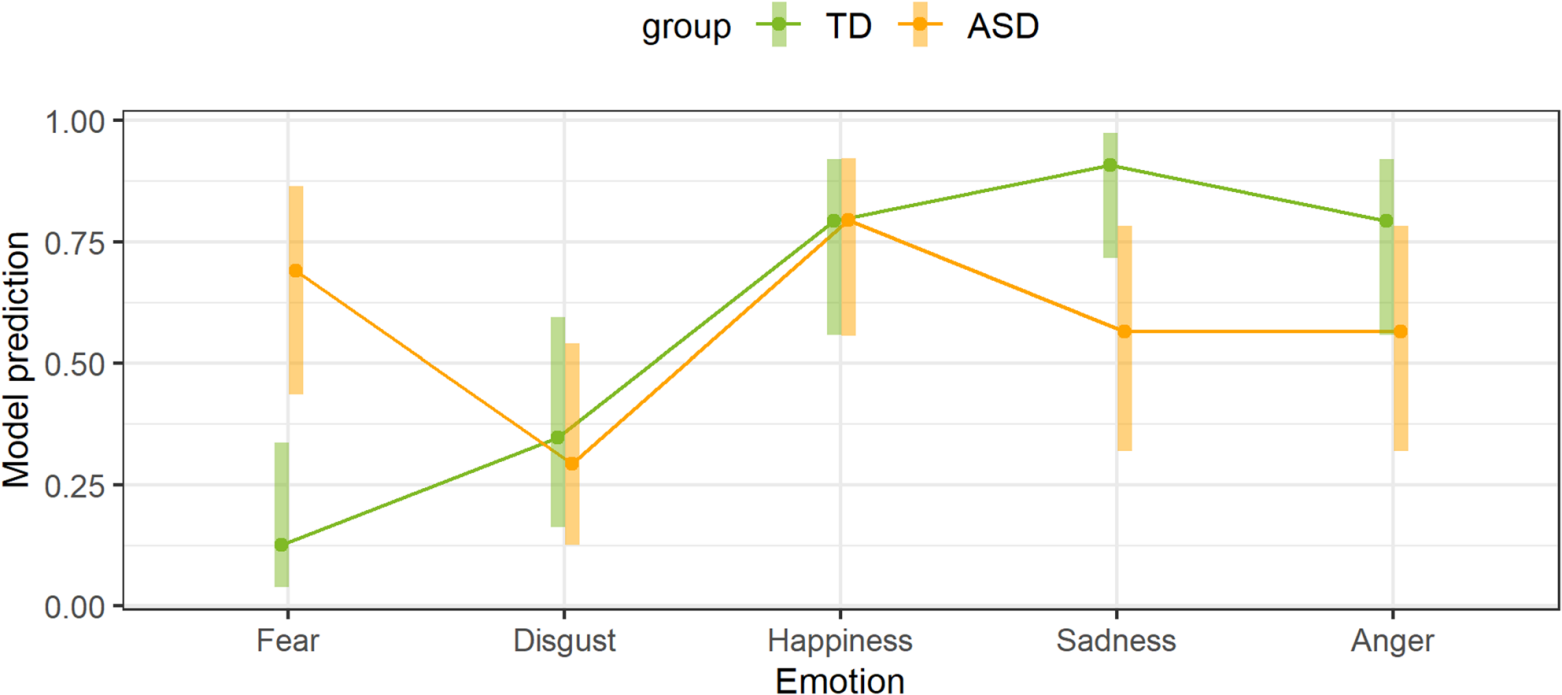
Model of emotion recognition

Figure 1 shows the model prediction for emotion recognition. When the ASD group was compared with the TD group, the model prediction was significant only for the emotion “fear” (OR = 15.41, 95%-KI: 1.72-138.14, *p* = .007). The model prediction for sadness had an OR of 0.13 (95%-KI: 0.01–1.24) and failed to reach significance (*p* = .098). In addition, the emotions of disgust (OR = 0.78, 95%-KI: 0.12–5.22, *p* = 1), happiness (OR = 1.01, 95%-KI: 0.13–7.85, *p* = 1) and anger (OR = 0.34, 95%-KI: 0.05–2.46, *p* = .8) were not significant.

The two groups mentioned a specific example of each emotion with varying frequency (see Supplement Figure S2). A specific example of the emotion “fear” was given by 71% of the TD group and by 50% of the ASD group. For disgust, 67% of the TD group and 42% of the ASD group were named a specific example. A specific example of the emotion “happiness” was given by 83% of the TD group and by 71% of the ASD group. For sadness, 75% of the TD group and 33% of the ASD group named a specific example. A specific example of the emotion “anger” was given by 79% of the TD group and by 54% of the ASD group.

**Fig. 2:**
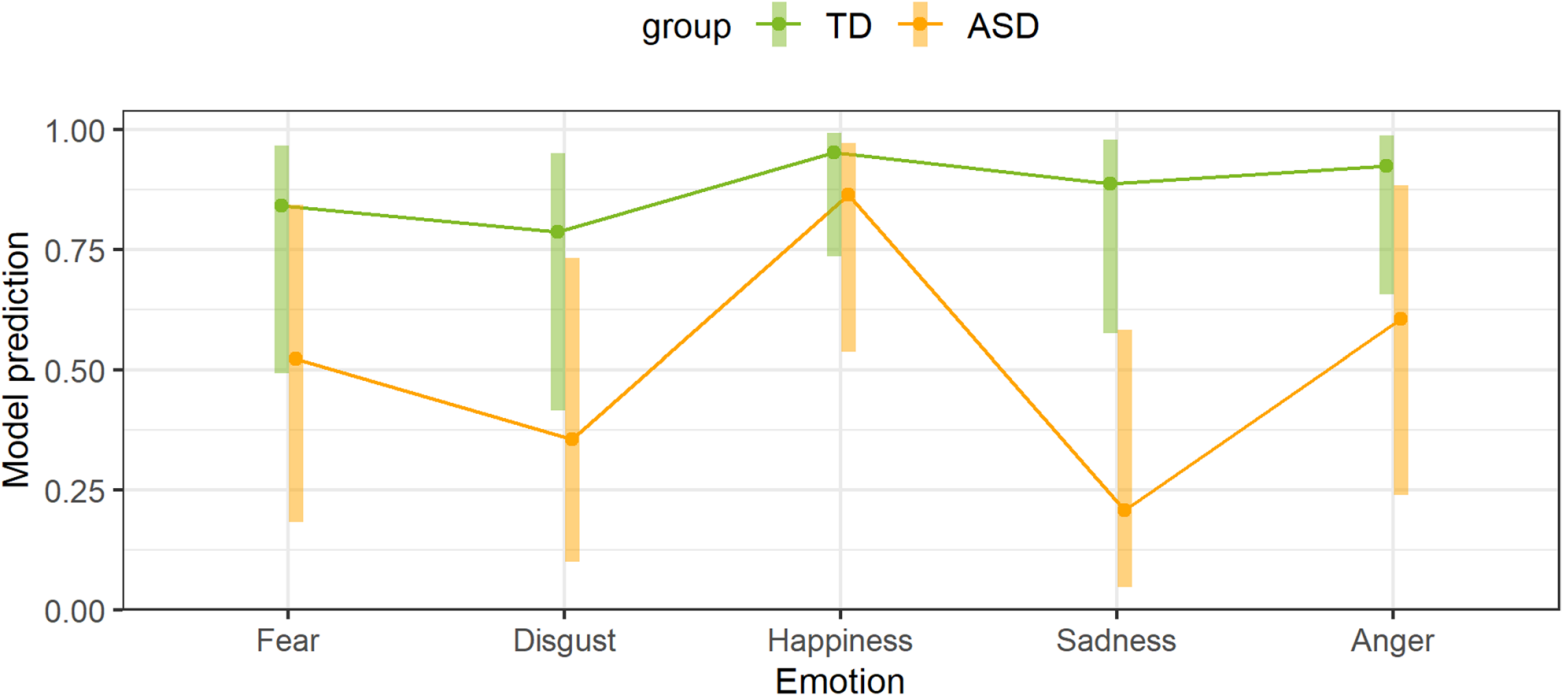
Model of experienced emotion examples

Figure 2 shows the model prediction for the experienced emotion example. When the ASD group was compared with the TD group, the model prediction was significant only for the emotion “sadness” (OR = 0.03, 95%-KI: 0.001–0.85, *p* = .034). The emotions of fear (OR = 0.21, 95%-KI: 0.01–4.28, *p* = .9), disgust (OR = 0.15, 95%-KI: 0.01–3.09, *p* = .529), happiness (OR = 0.32, 95%-KI: 0.01–8.53, *p* = 1) and anger (OR = 0.13, 95%-KI: 0.01–2.99, *p* = .462) were not significant.

For both emotion recognition and experienced emotion, a model for the ASD group was used to test whether the length of therapy had an effect on emotion tasks. No significant effects were found for either emotion recognition or experienced emotion (*p* > .05).

**Fig. 3:**
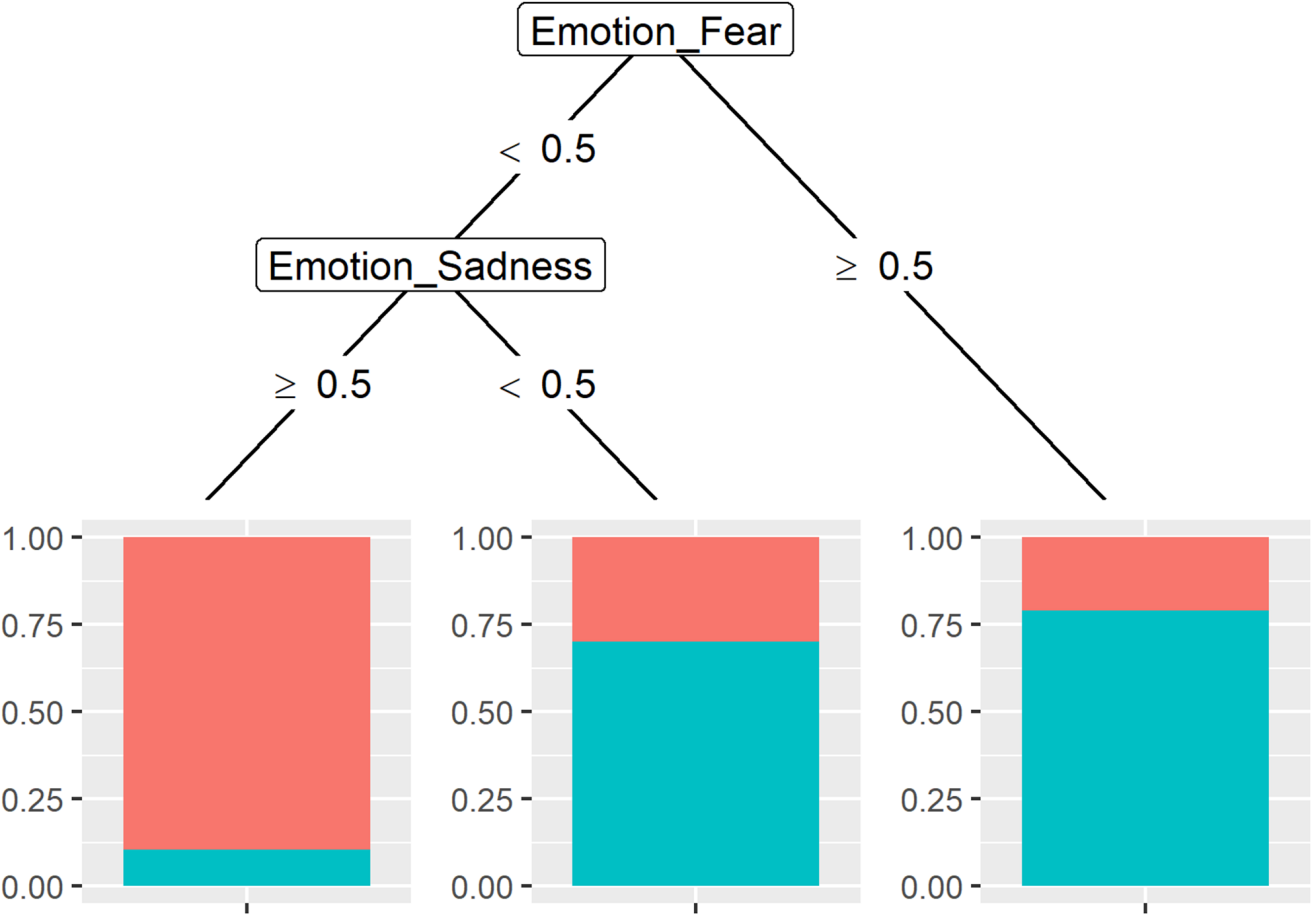
Decision tree for the emotion task. *Note.* < 0.5 = Emotion not recognized, ≥ 0.5 = Emotion correctly recognized; red = TD, blue = ASD

The predictor variables age, recognition of the respective emotion, and the specific naming of a situation in which the respective emotion was experienced were included to predict the allocation to the respective group (TD or ASD). Only the emotion recognition of fear and the emotion recognition of sadness were relevant for classification (see Fig. 3). Of the children who correctly recognized the emotion “fear”, just over 75% have ASD. Of those who did not recognize “fear” or “sadness”, just under 75% have ASD. Among those who did not recognize “fear” but correctly recognized “sadness”, over 87.5% are TD children. The accuracy was 81.25%, the sensitivity was 91.67%, and the specificity was 70.83%.

### Visual preference task

The percentage fixation time varies according to group, medium, and presentation (see Supplement Figure S3). For the medium image, there are 144 observations per group and presentation because each child saw six images in each presentation. For video, there are 96 observations because each child saw four videos in each presentation. For the non-social image, the mean duration of gaze fixation was 29.97% (*SD* = 17.73) of the total frames in the TD group and 27.13% (*SD* = 18.34) in the ASD group. For social image, the mean duration of gaze fixation was 52.12% (*SD* = 21.29) in the TD group and 45.74% (*SD* = 23.48) in the ASD group. For the non-social video, the mean duration of gaze fixation was 31.21% (*SD* = 25.47) for the TD group and 36.33% (*SD* = 26.48) for the ASD group. For the social video, the mean duration of gaze fixation was 53.17% (*SD* = 28.24) for the TD group and 40.84% (*SD* = 26.86) for the ASD group.

**Fig. 4:**
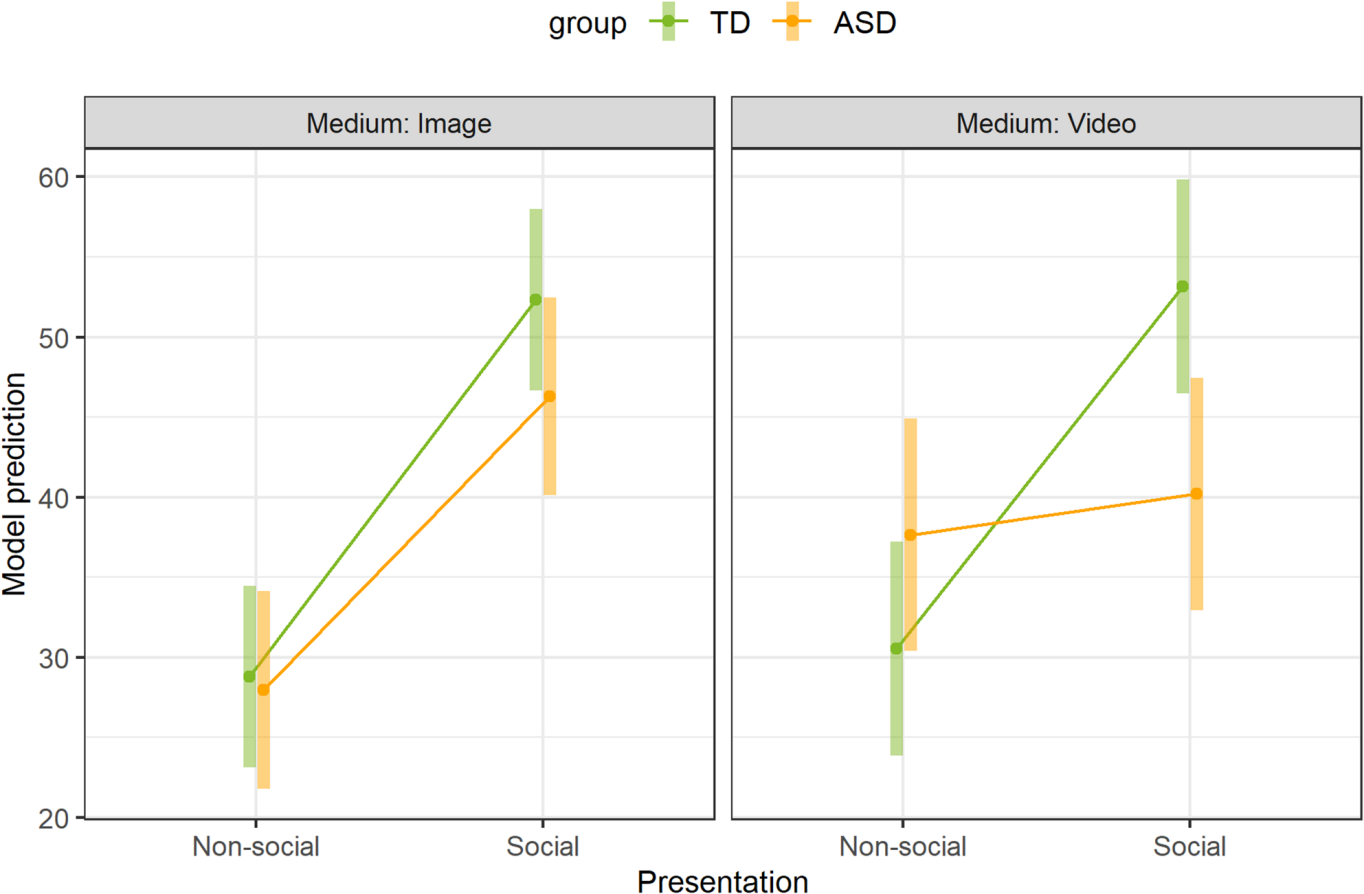
Model predictions of the duration of gaze fixation for the combination of group, presentation, and medium

The linear mixed model with gaze fixation duration as the dependent variable, main effects and interactions between (1) group, presentation, and medium, (2) medium, presentation, and FSK, and between (3) group, presentation, and age and simultaneous random variation between children and stimulus pairs showed no significant results (*p* > .05). The post-hoc tests revealed no significant results for differences between groups for each combination of presentation and medium or for the medium for each combination of group and presentation (*p* > .05). In terms of differences in presentation for each combination of group and medium, there were no significant differences between the social and non-social only in the ASD group with the video as the medium (*p* > .05). Both the post-hoc test for differences between the presentation in the ASD group and the medium image (OR = 18.36, 95%-KI: 9.0-27.7, *p* < .001) and the post-hoc test for differences in the TD group and the medium image (OR = 23.55, 95%-KI: 15.06-32.0, *p* < .001) as well as the medium video (OR = 22.64, 95%-KI: 12.27-33.0, *p* < .001) were statistically significant. Figure 4 shows model predictions for the combination of group, presentation, and medium.

The number of gaze changes between groups varies according to the medium (see Supplement Figure S4). For the medium image, the mean number of gaze changes was 5.49 (*SD* = 4.2) in the TD group and 5.58 (*SD* = 4.24) in the ASD group. For the medium video, the mean number of gaze changes was 7.84 (*SD* = 7.04) in the TD group and 5.37 (*SD* = 4.97) in the ASD group.

**Fig. 5:**
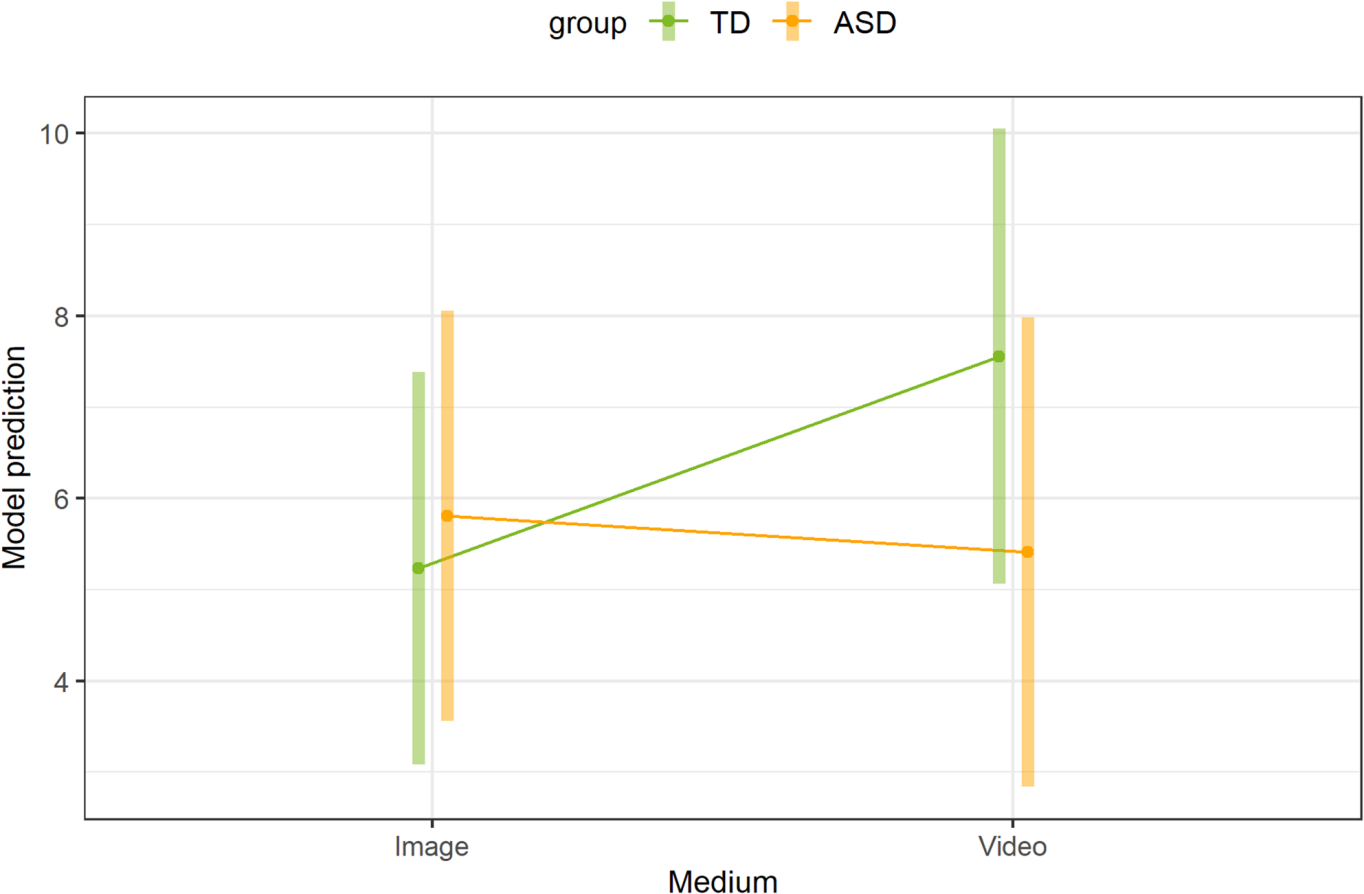
Prediction model of the number of gaze changes for the combination of group and medium

Figure 5 shows the prediction model of the number of gaze changes for the combination of group and medium. The model prediction between the ASD and TD groups was not significant for either the medium image (OR = 0.57, 95%-KI: -2.55-3.70, *p* = 1) or the medium video (OR = -2.15, 95%-KI: -5.38-1.09, *p* = .27).

**Fig. 6:**
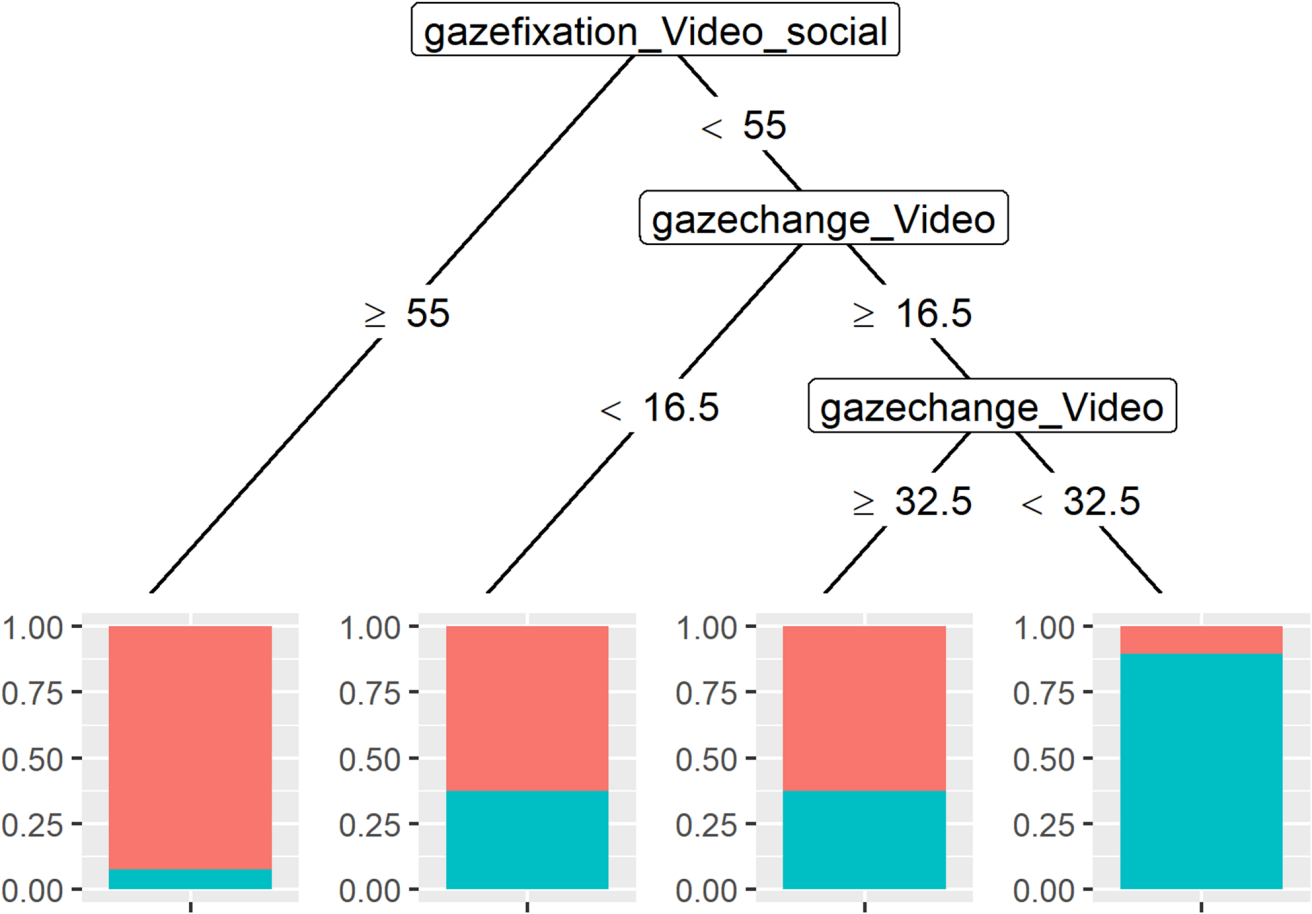
Decision tree for the visual preference task. *Note*. red = TD, blue = ASD

The model was also tested to determine whether there were any differences between each image and video pair. However, no significant results were found (*p* > .05). Therefore, only the predictor variables age, duration of gaze fixation on images and videos, social and non-social, and number of gaze changes on images and videos were used in the decision tree to predict group assignment (TD or ASD). Only the duration of gaze fixation on social videos and the number of gaze changes on videos were relevant for classification (see Fig. 6). Of the children in the group who had a relative duration of gaze fixation of more than 55% on the social video, just over 87.5% were TD children. Of those who had less than 55% of their gaze fixation on the social video and changed their gaze less than 16.5% on the medium video, just under 62.5% were TD children. This is also the case for those who changed their gaze on the medium video by more than 32.5%. Among children with less than 55% relative gaze fixation on the social video and a gaze change of between 16.5% and 32.5% on the medium video, just over 87.5% had ASD. The accuracy was 81.25%, the sensitivity was 70.83%, and the specificity was 91.67%.

### Comparison of the decision trees

In the visual preference task, 29 children were predicted to be in the TD group, and 19 children were in the ASD group (see Table 1). For the emotion recognition task, the group assignment was conducted in the reverse order (see Table 1). When the two predictions were compared, 16 children were assigned to the TD group, and 16 children were assigned to the ASD group in both tasks. In the emotion recognition task, 13 children were assigned to the ASD group, who were assigned to the TD group in the visual preference task. In addition, three children were assigned to the ASD group in the visual preference task but were assigned to the TD group in the emotion recognition task.

**Table 1:** Correct allocation to the groups in relation to the two tasks

Emotion recognition task				Visual preference task			
		Allocation				Allocation	
		TD	ASD			TD	ASD
**Correct allocation**	TD	17	7	**Correct allocation**	TD	22	2
	ASD	2	22		ASD	7	17

The predictions for both tasks were then compared in relation to actual group membership (see Table 2). Out of the actual TD group of 24 children, 16 children were correctly classified by the predictions of both tasks. A further six children were correctly assigned to the TD group by the visual preference task but incorrectly assigned to the ASD group by the emotion recognition task. In contrast, one child was correctly assigned to the TD group by the emotion recognition task and incorrectly assigned to the ASD group by the visual preference task. One child was incorrectly assigned to the ASD group for both tasks. From the actual ASD group, which also consisted of 24 children, 15 children were correctly classified by the predictions of both tasks. Another two children were correctly assigned to the ASD group by the visual preference task but incorrectly assigned to the TD group by the emotion recognition task. In contrast, seven children were correctly assigned to the ASD group by the emotion recognition task and incorrectly assigned to the TD group by the visual preference task. Overall, there was no child who was incorrectly assigned to the TD group on both tasks.

**Table 2:** Comparison of the correct allocation to the groups in relation to the two tasks

TD				ASD			
		Emotion recognition task				Emotion recognition task	
		TD	ASD			TD	ASD
**Visual preference task**	TD	16	6	**Visual preference task**	TD	0	7
	ASD	1	1		ASD	2	15

In summary, the prediction on the emotion recognition task correctly classified 17 TD children and 22 children with ASD. Prediction on the visual preference task had correctly classified 22 children with ASD and 17 TD children.

## Discussion

Due to the long waiting period and the associated uncertainty until a diagnosis of ASD is established, we investigated whether tablet-based digital screening with emotion recognition and visual preference tasks could discriminate between children with ASD and children without ASD.

### Emotion recognition task

Previous studies have shown mixed results concerning whether children with ASD are less accurate at recognizing basic emotions than TD children are [7, 31, 36]. Our results support the findings of Evers et al. [38] that children with ASD select the emotion “fear” more often than TD children do. However, while in Evers et al. study [38] dynamic facial expressions were used, in our study static facial expressions were used. However, the finding by Evers et al. [38] was no longer significant after correction for response bias. Our study indeed confirms a significant difference in the recognition of the emotion “fear”. The children in the ASD group were significantly more likely to correctly identify the emotion “fear”. This contradicts the results of Griffiths et al. [40], who reported a generally low accuracy of emotion recognition for fear in both groups. In our study, the TD group identified the emotion “fear” least correctly but identified the emotion “sadness” most frequently. However, the difference in emotion recognition of sadness between the groups did not reach significance. One possible reason for this difference is that the Griffith et al. study [40] used images of children and adults expressing emotions at varying levels of intensity. By contrast, our study used a single image per emotion of a child displaying a single (high) level of intensity. Additionally, there were no differences between the groups in the recognition of the emotion “happiness” in our study. Both groups were able to recognize this emotion equally well and could therefore be used as a baseline or control variable.

Overall, the TD group reported a specific example of a situation in which the emotion was experienced more often than the ASD group did. This descriptive finding may support findings by Huggins et al. [45] that individuals with ASD have difficulty recognizing and understanding their own emotions. However, only the difference in naming a specific situation for the emotion “sadness” was significant. Thus, it appears that children with ASD generally have difficulties with the emotion “sadness”, both in recognizing it and in naming a specific example. Although it is well-known that people with ASD have difficulty recognizing emotions, this may not be the only reason for the observed differences between the TD and ASD groups. This task may require more complex cognitive processing than pure emotion identification because asking for an example requires a precise understanding or reconstruction of a (possible) context. For example, in a social film experiment, Hochhauser et al. [87] were able to show that individuals with ASD were less able to consider different protagonists quickly and flexibly than individuals with TD, and that this was connected to the perspective-taking difficulties of the participants with ASD. The group difference in the present task could indicate that the item ‘give an example’ requires more complex processes, such as perspective taking, visual attention, and language production, and thus indicates more difficulty than pure emotion recognition.

To assign children to either the ASD or TD group via the emotion recognition task, it seems sufficient to use only emotion recognition of fear and sadness. If the children recognized the emotion “fear” correctly, they were assigned to the ASD group. If the children do not correctly recognize the emotion “fear”, they are checked to see if they correctly recognize the emotion “sadness”. If they do not, they are once more assigned to the ASD group. If they recognize the emotion “sadness” correctly, they are assigned to the TD group. With an accuracy of 81.25%, a sensitivity of 91.67% and a specificity of 70.83%, the diagnostic accuracy is quite good.

### Visual preference task

Many studies have shown that children with ASD, often under six years of age, have a significantly greater preference for non-social stimuli than for social stimuli [54, 55]. However, this result was not observed in our study of 6- to 11-year-olds. Here, the relative duration of gaze fixation was significantly greater for social images than for non-social images in both groups. However, a study by Matyjek et al. [62] showed that in adulthood, people with ASD no longer preferred non-social stimuli over social stimuli. Instead, the difference between the ASD and TD groups is that people with ASD do not discriminate between the presentations, whereas a difference occurs in the TD group. Our results support this interpretation, as we found a significant difference for the video medium in the TD group, i.e., the relative duration of gaze fixation for the social video was significantly greater than that for the non-social video. In contrast, no difference was found in the ASD group. The different visual preference results observed in toddlers, children, and adults, both with and without ASD, suggest potential changes in diagnostic patterns over time. However, due to the lack of longitudinal data, it is not possible to draw any conclusions about how the significance of symptoms is represented over the course of a child’s development.

Additionally, no differences were found in this study in terms of the frequency of gaze changes. However, the change in gaze in the medium video based on the decision tree seems to be a predictor – together with the percentage of time spent watching the social video – for the classification of the groups. If the children had a relative duration of gaze fixation greater than 55% on the social video, they were assigned to the TD group. If the relative duration of gaze fixation was less than 55% and they changed their gaze less than 16.5% on the video, they were also assigned to the TD group. This was also the case if their gaze shifts were above 32.5%. If their gaze shifts were between 16.5% and 32.5%, they were assigned to the ASD group. With an accuracy of 81.25%, a sensitivity of 70.83%, and a specificity of 91.67%, the diagnostic accuracy is promising. 

### Task validity for clinical use

Overall, the prediction accuracy of the models of the two tasks is 81.25%. In the emotion recognition task, 17 out of 24 TD children and 22 out of 24 children with ASD were correctly predicted. In the visual preference task, the results were reversed. This suggests that both tasks can be used for digital screening, and it is appropriate to combine them, as they are complementary. We conclude that digital screening can be implemented by existing digital technologies and that selected emotion recognition and visual preference tasks can discriminate between children with and without ASD.

### Limitations and implications

However, it is important to note that the predicted values apply only to this sample and should not be generalized. Another limitation is the sample size of only 24 children per group (ASD and TD), although this is a typical sample size for clinical or ASD studies and is large enough for analysis. However, this small sample size increases the risk of reduced statistical power and model instability. Similarly, the number of predictor variables increases the risk of overfitting the model. Therefore, the results should be interpreted with caution. Notably, not all diagnosed children in the ASD group met the threshold for suspected ASD on the parent-completed FSK (the FSK score was negatively correlated with the duration of therapy). However, this was accounted for in the analyses and had no significant effect. Thus, the two tasks appear to be independent of any therapy received to date, which is a strength in itself. However, this is surprising in regard to the emotion recognition task, given that emotion recognition is often addressed in therapy. Currently, there is no information available regarding the therapy that has been provided to the children. It is possible that emotion recognition has not yet been addressed in therapy. Overall, the task of emotion recognition has two methodological limitations. Firstly, the images only depict female individuals, even though the participants were male. This restricts the generalizability of the results, given that Hoffner [88] reported that people tend to identify more with people of the same gender and find them more attractive. Secondly, the single representation of each emotion restricts variability and may not capture the full range of expressions associated with it. This could reduce the robustness of the task.

The composition of the sample is also a limitation. The sample consists only of boys, which means that the results cannot be extrapolated to girls or other genders. This is also a typical sample for an ASD study, as there is an assumption that the phenotype of female autism is qualitatively different from that of male autism [66]. The study by Burrows et al. [89] indicated that girls demonstrated milder symptoms than boys. In addition, children with ASD and intellectual impairment were not included in this study, as children with ASD and intellectual impairment are more likely to be diagnosed than are children with ASD without intellectual impairment [13]. Therefore, digital screening is particularly needed for the group of children suspected of having ASD without intellectual impairment. It should be emphasized that the level of accuracy is high in both tasks, despite the presence of additional diagnoses in both groups. The results may be impacted by comorbidities. It would be interesting to investigate whether accuracy improves when children in the TD group do not have a diagnosis and children in the ASD group only have an ASD diagnosis. It is possible that the contradictory results of combining the two tasks are due to the additional diagnoses, such as ADHD. This should be verified.

A technical limitation of the visual preference task is that – because the tablet’s camera was located on the left side –, eye tracking worked better on the left side of the screen than on the right (see [60]). Therefore, a tablet with a camera in the center should be used in further investigations and will be implemented in future tests of the IDEAS project.

Despite the above limitations, our study has several strengths. In our study, we reported the sensitivity and specificity of typical discrimination tasks described in the ASD literature. The results are based on small yet adequate sample sizes, which are typical of ASD research. For the analyses, we used an advanced statistical model class of decision trees to estimate sensitivity and specificity, which represents the state of the art in statistical analysis and controls for the influence of therapy duration.

To replicate these study results and verify the accuracy of the prediction, a new sample should be collected in the future. The sample size should also be increased in future studies. As the long-term goal is to contribute to the early identification of ASD, this study should be repeated with younger children. In Germany, ASD is diagnosed at an average age of 6;6 years [13]. When testing younger children, therefore, there may only be a suspicion, rather than a diagnosis. In this case, it would be necessary to check whether children identified through the tests were later diagnosed with ASD. In terms of potential gender differences, future studies should include girls with and without ASD, boys with and without ASD, and mixed-gender groups with and without ASD. It is important to conduct the study with both girls with and without ASD. For example, a study by Burrows et al. [89] showed that the gender ratio was balanced within the group classified as being at high risk of ASD. Burrows et al. [89] conclude that girls may be underdiagnosed because their symptoms are more subtle and cannot be adequately captured by current diagnostic tools. Therefore, it would be worthwhile to investigate whether the tasks used in this study can also identify girls with ASD. Moreover, the target group of the ASD group consists of children with ASD without intellectual impairment who can communicate verbally. It would be interesting to conduct a similar study with children with ASD with intellectual disability who communicate via augmentative and alternative communication (AAC) to investigate whether a digital screening based on the visual preference task might also be appropriate for children who use AAC, as no verbal speech is needed, only gaze.

Our study shows promising results that may facilitate the identification of children with ASD without intellectual impairment in the future. New task formats may be considered for this purpose. As the emotion recognition task is currently administered verbally, future studies should test whether the task works at least as well when children are asked to select the emotion emoji instead of naming the emotions. Mimicry tasks should also be tested in the future. Studies have shown that mimicry is lower in people with ASD and that the differences between groups in terms of mimicry can also occur when individuals interact with avatars [90]. In this context, Dotzer et al. [91] investigated whether facial expression recognition (FER) reliably detects smile events and whether facial expressions can thus be digitally and automatically recorded and scored. The result was that the FER reliably recognized smile events and corresponded to a human evaluation [91]. This could therefore be transferred to the emotion of happiness and should be verified for other basic emotions.

## Conclusion

Ultimately, digital automated screening might promote (in future) earlier detection of ASD. However, it is not intended to replace diagnostics but can support clinicians by objectifying a step of the decision-making process, reducing the burden of clinical practice, and providing a resource (cf [21]). Relief could be provided by using objective digital screening to remove children who are not suspected of having ASD from the waiting list for the diagnosis of ASD, thereby reducing the number of children on the waiting list. Digital screening can therefore be a resource and a relief for clinicians and families with suspected cases of ASD.

## Supplementary Information

Below is the link to the electronic supplementary material.

Supplementary Material 1

## Data Availability

The data will be shared upon reasonable request to the corresponding author with the permission of the entire consortium (Fraunhofer Institute for Digital Media Technology; KIZMO GmbH; SpeechCare GmbH; University of Leipzig Medical Center​; TU Dortmund University).

## Footnotes

1 The project IDEAS (Identification of Autism Spectrum Disorder using speech and facial expression recognition) was funded by the German Federal Ministry of Research, Technology and Space (BMFTR; funding code: 13GW0584A-E).

2 Social attention refers to individuals paying preferential attention to social stimuli, whereas ‘visual preference’ is a general term for the preference of either social or non-social stimuli.

## Abbreviations

AAC: Augmentative and alternative communication
AD(H)D: Attention-deficit (hyperactivity) disorder
ASD: Autism spectrum disorder
EAST: Early autism screening tool
FER: Facial expression recognition
FSK: German version of the Social Communication Questionnaire
IDEAS: Identification of Autism Spectrum Disorder using speech and facial expression recognition
SACS: Social Attention and Communication Surveillance
TD: Typically developing
WIN4ASD: Web Italian Network for Autism Spectrum Disorder
