# Supplementary material for: Digital screening of children with ASD: diagnostic accuracy of emotion recognition and visual preference tasks
Source: BMC Psychiatry. 2025 Dec 25;26:75. doi: 10.1186/s12888-025-07725-z (PMC12849604; doi:10.1186/s12888-025-07725-z)

Supplement

Figure S1

Emotion recognition

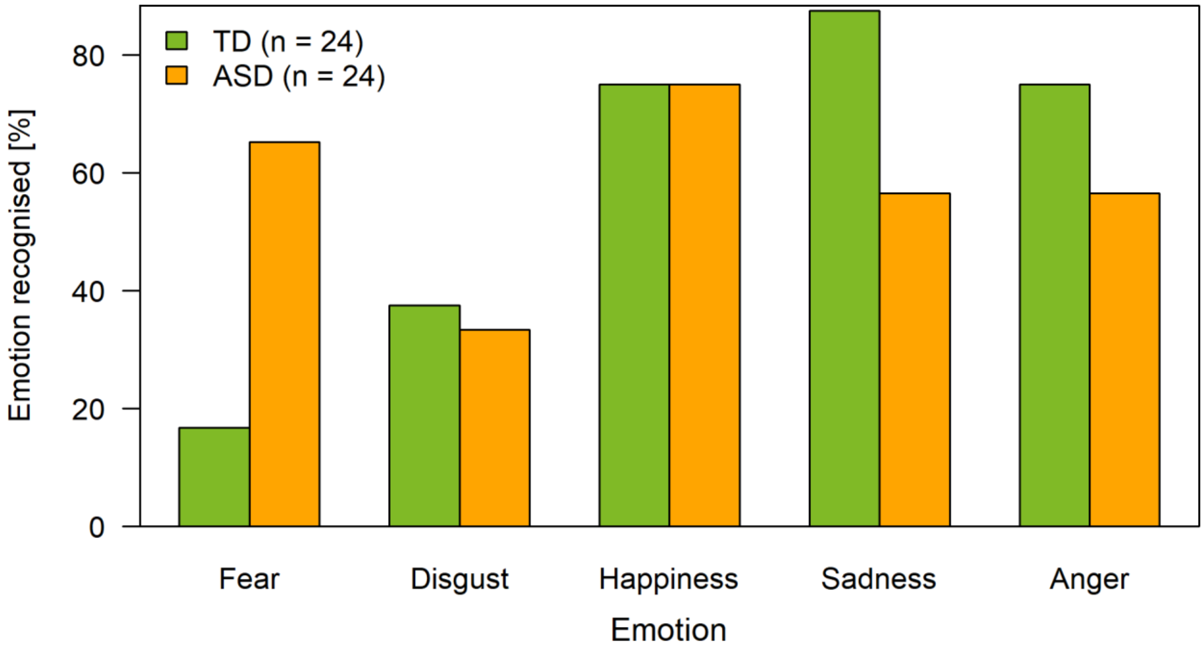

Supplement

Figure S2

Examples of experienced emotions

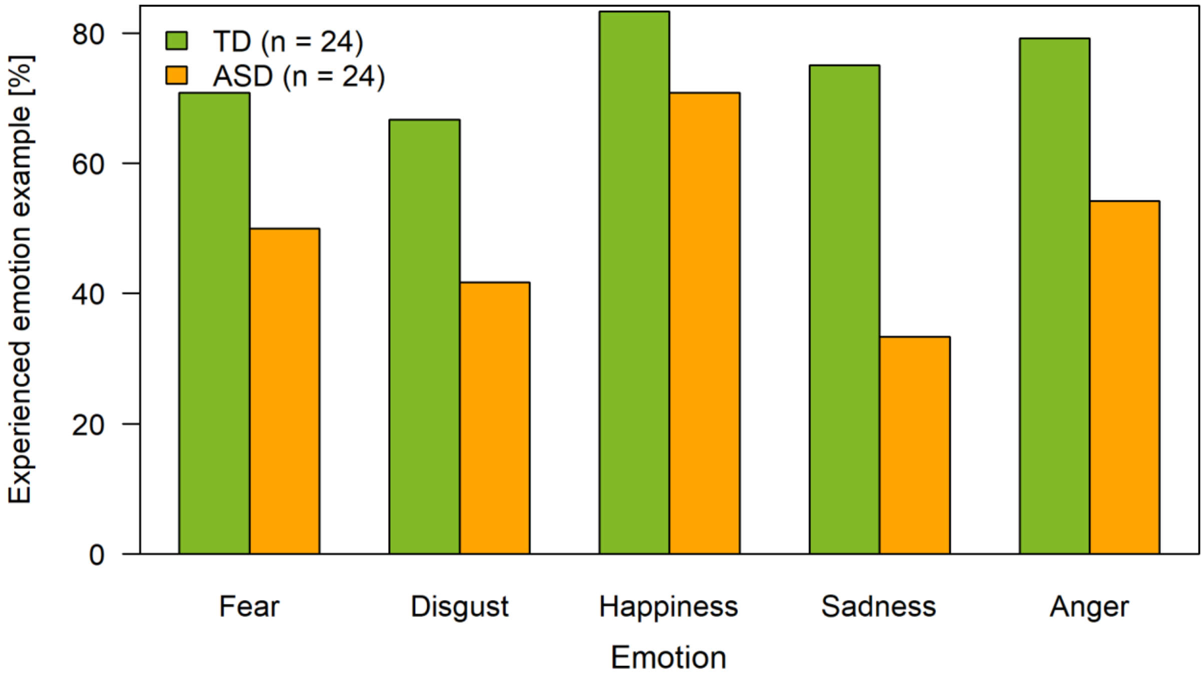

Supplement

Figure S3

Duration of gaze fixation in relation to group, presentation, and medium

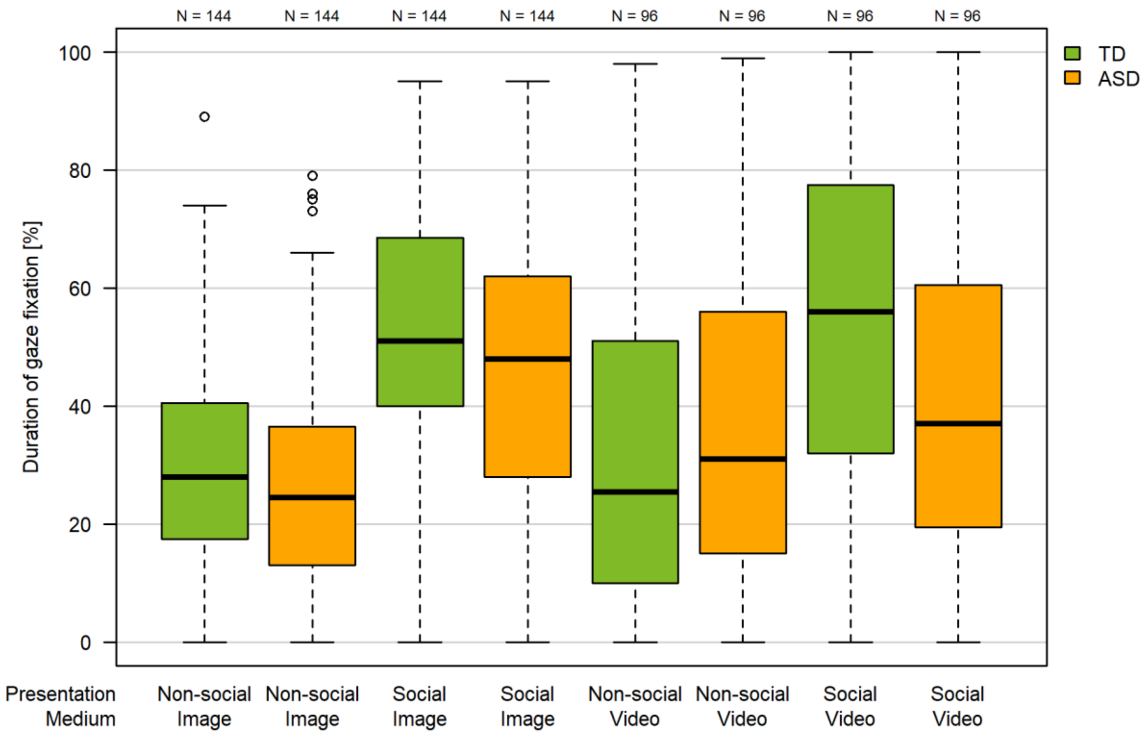

Supplement

Figure S4

Number of gaze changes between groups according to the medium

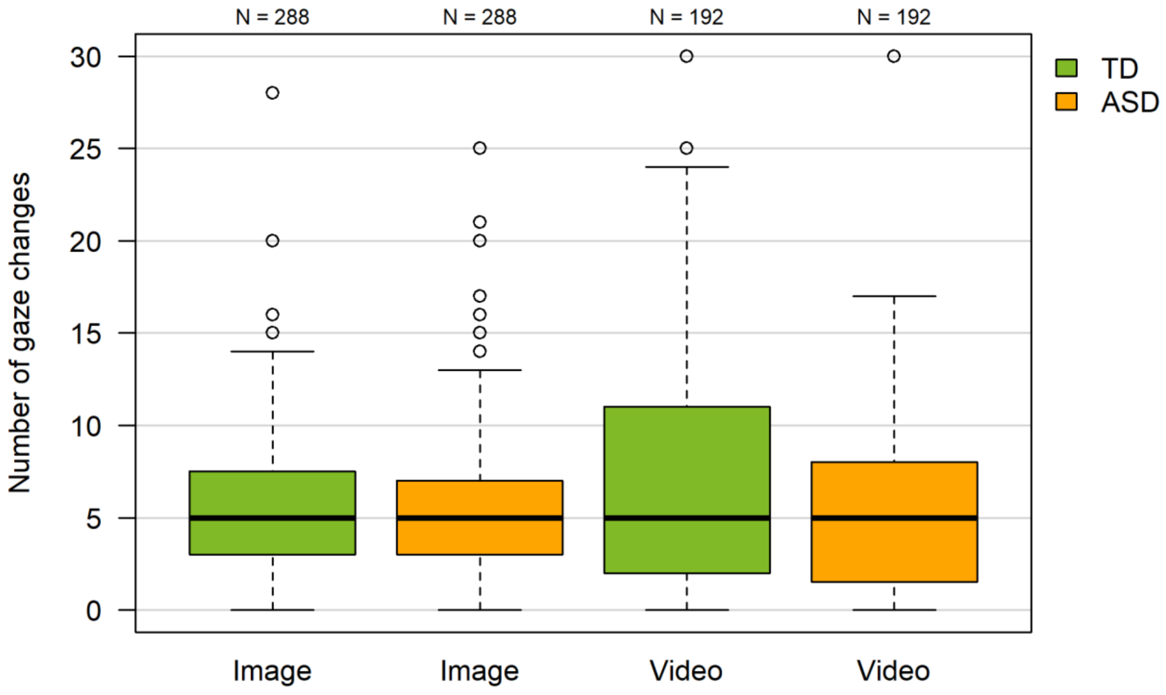

Supplement: Supplementary file 1 — Supplementary Material 1 [file 12888_2025_7725_MOESM1_ESM.pdf]
